# Supplementary material for: Synchronization in collectively moving inanimate and living active matter
Source: Nat Commun. 2023 Sep 13;14:5633. doi: 10.1038/s41467-023-41432-1 (PMC10499792; doi:10.1038/s41467-023-41432-1)
Supplement: Supplementary file 1 — Supplementary Information [file 41467_2023_41432_MOESM1_ESM.pdf]

SUPPLEMENTARY INFORMATION

Synchronization in collectively moving inanimate and living active matter

Michael Riedl[1,*], Isabelle Mayer[1], Jack Merrin[1], Michael Sixt[1,*] and Björn Hof[1,*],

[1] Institute of Science and Technology Austria (IST Austria), Klosterneuburg, Austria.

[*] Correspondence: e-mail: michael.riedl@ist.ac.at; sixt@ist.ac.at, bjoern.hof@ist.ac.at

(Dated: February 1, 2022)

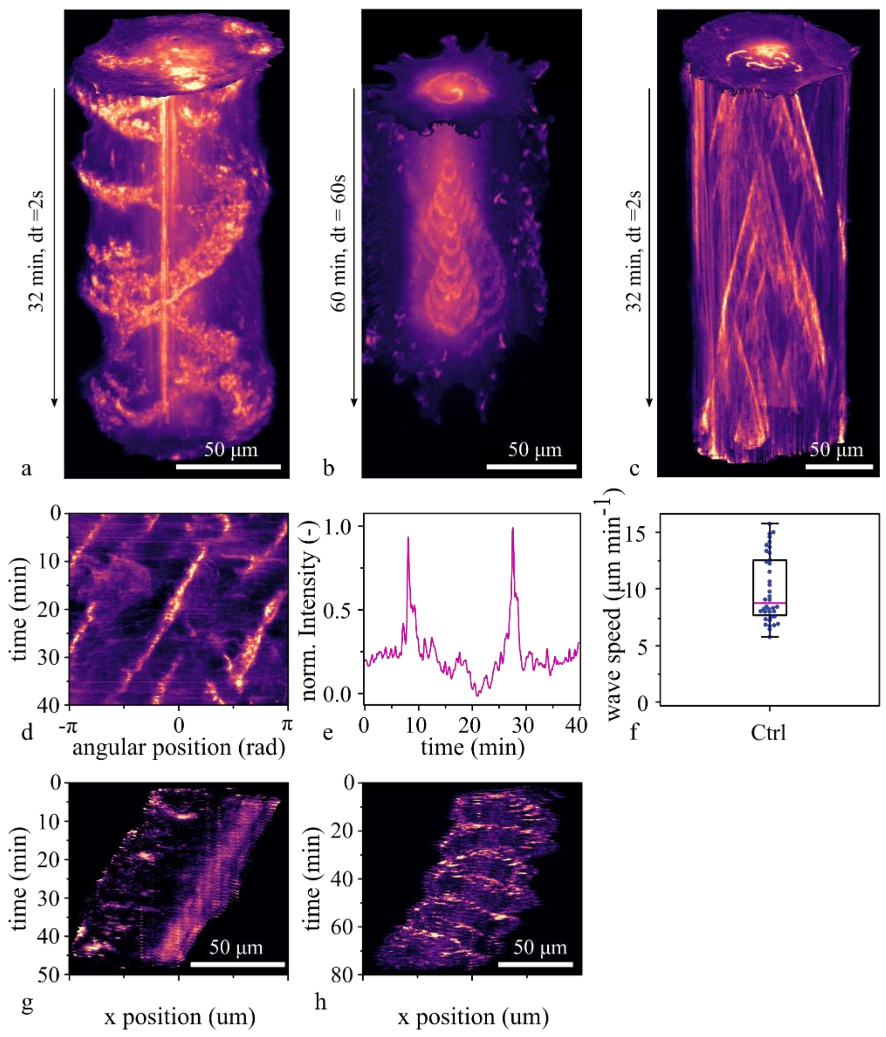

Supp. Fig. 1 | Polymerizing actin waves characterization in single cells on circular and line patterns.

a,b,c, Space-time representations of different manifestations of the observed actin polymerization waves. While in **a** multiple persistent waves travel orderly through the cell, in **c**, small short-lived wavelets form and propagate through the cell. In **b**, we observe spiral wave formation reemerging from the same initial point. **d,** The polar transformed and sliced space-time representation of the wave dynamics corresponding to **a**. Here, up to 3 waves are present simultaneously and annihilate upon collision. **e,** Extracting the intensity along fixed angular position results in peaks corresponding to actin polymerization waves passing through. **f,** Actin polymerization wave propagation speed within stationary cells in circular confinement. The bottom and top of each box represent the first and third quartiles, respectively, while the band inside the box marks the median. Whiskers extend to 1.5 times the interquartile range. **g, h,** Space-time representation of a single cell migrating on a line pattern (width = 50 µm). The polymerizing actin waves at higher **g**, and lower **h,** frequencies.

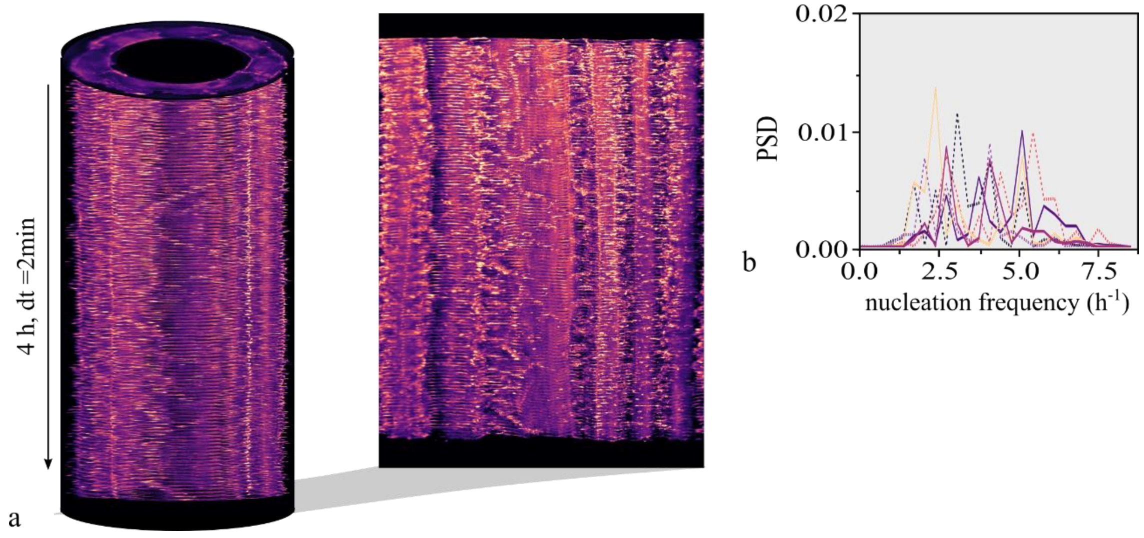

Supp. Fig. 2 | Absence of frequency locking and collective rotation.

An example of 7 cells on an adhesive ring pattern (diameter = 300 μm, width = 35 μm) that do not collectively migrate. **a,** The space-time representation and the corresponding polar transformation omits collective rotation or convergence of nucleation frequencies of polymerizing actin waves. **b,** The power spectrum density plot omits a common dominant frequency across the collective, despite the presence of cell-specific frequency maxima.

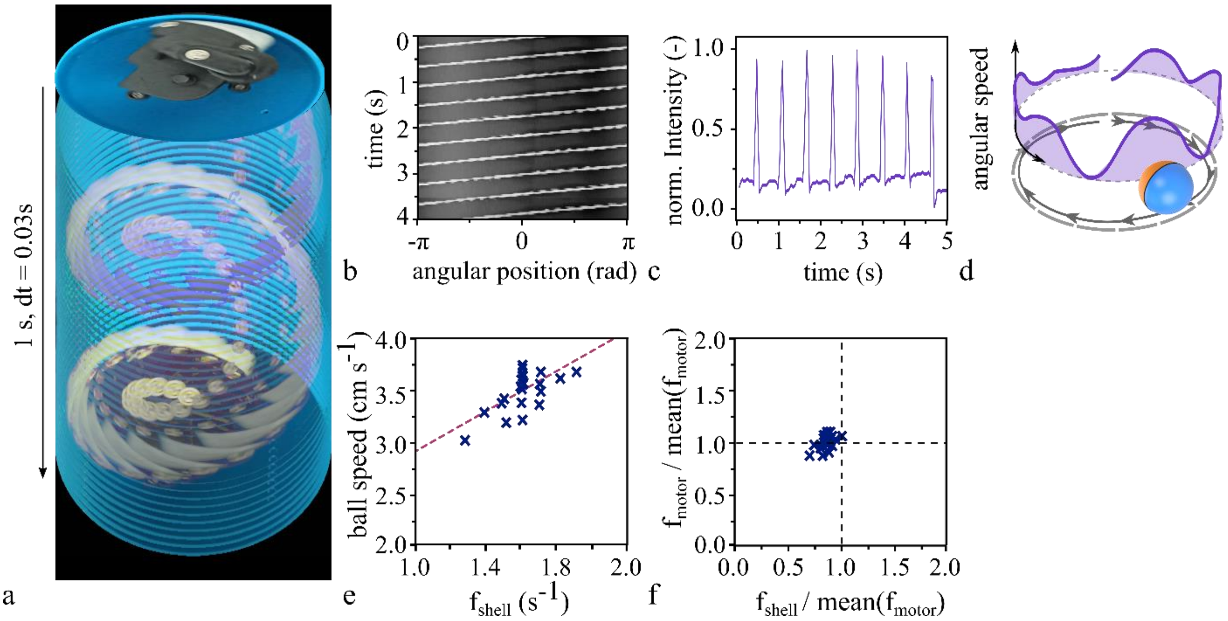

Supp. Fig. 3 | Single motorized ball characterization.

a, The space-time representation visualizes the rotating motion of the motor within the fixed shell. **b,** The position of the internal motor and weight periodically rotates in the fixated state around its axis. **c,** The extracted intensity peaks correspond to the revolutions. **d,** An example trajectory of a rotating ball shows the corresponding characteristic oscillations in speed along the boundary of the confinement (D = 0.2 m). **e,** The frequencies extracted from velocities of single balls rolling in a circular confinement show the non-uniform distribution (D = 0.5m, n =25). **f, T**he rotation frequencies of the shells correspond to a negative shift of the motor frequencies. Both quantities were normalized with the mean motor frequency.

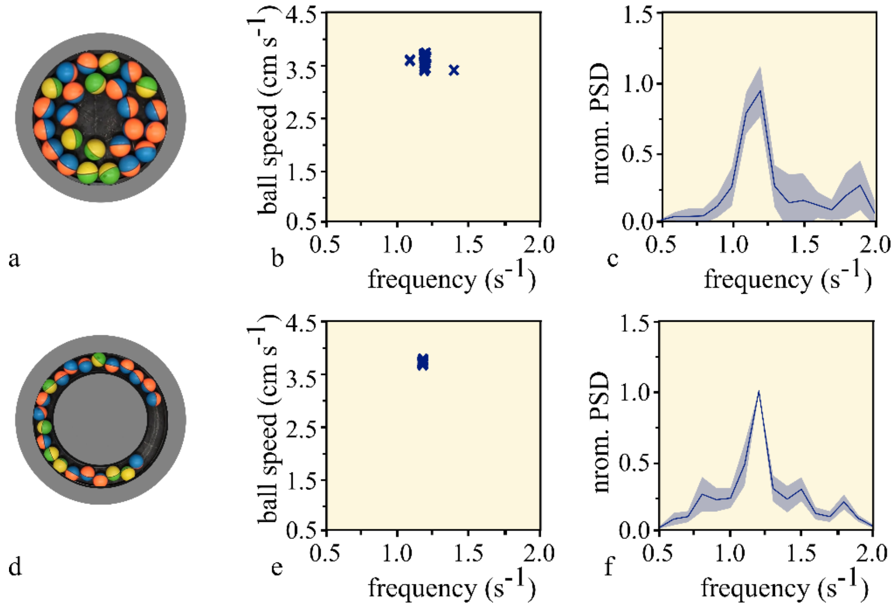

Supp. Fig. 4 | Rotation frequency characterization in collectives of motorized balls in circular and annular confinements.

a,**d**, Snapshot of the ordered state in a **a**, circular confinement (D = 50 cm, n = 25) and **d**, ring confinement (D_{outer} = 75 cm, D_{inner} = 50 cm, n = 22). **b**,**e**, Frequency maxima in the ordered, collectively rotating state **b**, circular and **e**, ring confinement. **Fig. 2b**. shows the frequency of individual balls. During the ordered rotation, the individually spread frequency lock to a single collective frequency. **c**,**f**, The corresponding frequency spectrum density for the collectively rotating **c**, circle, and **f**, ring.

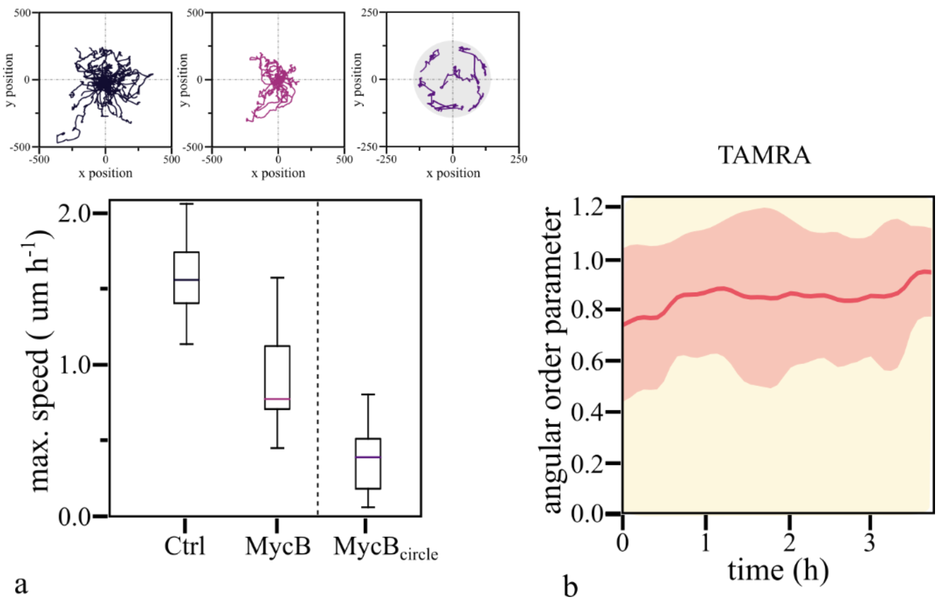

Supp. Fig. 5 | Control experiments regarding the applied drug treatments underline the specificity for our purpose.

a, MycB treated cells cease to migrate efficiently, their maximal speed is strongly reduced and fail to establish collectivity in circular confinements (D=159 um, n=8). Above we show sample trajectories for each condition respectively and over a time window of 4 h. While unconfined endothelial cells migrate randomly, when treated with MycB their migration behavior mimics a Brownian particle and the explored space is strongly reduced. In confined conditions a population of treated cells exhibit no collective migration and individuals reside at one location. **b**, The TAMRA-stained cell populations establish collective rotation in circular confinements (D = 150 um, n = 10) (Mean ± SD).
