# Supplementary material for: Synchronization in collectively moving inanimate and living active matter
Source: Nat Commun. 2023 Sep 13;14:5633. doi: 10.1038/s41467-023-41432-1 (PMC10499792; doi:10.1038/s41467-023-41432-1)
Supplement: Supplementary file 3 — Description of Additional Supplementary Files [file 41467_2023_41432_MOESM3_ESM.pdf]

Description of Additional Supplementary Files

Supplementary Movie 1 Pattern of polymerizing actin

Supplementary Movie 2 Actin polymerization waves in cells on adhesive line pattern

Supplementary Movie 3 Collective migration of cells

Supplementary Movie 4 Collective migration of motorized balls

Supplementary Movie 5 Destabilization Experiments
